# A direct comparison of unconscious face processing under masking and interocular suppression

**DOI:** 10.3389/fpsyg.2014.00659

**Published:** 2014-07-07

**Authors:** Gregory Izatt, Julien Dubois, Nathan Faivre, Christof Koch

**Affiliations:** ^1^Computation and Neural Systems, California Institute of TechnologyPasadena, CA, USA; ^2^Laboratory of Cognitive Neuroscience, Brain Mind Institute, School of Life Sciences, École Polytechnique Fédérale de LausanneLausanne, Switzerland; ^3^Allen Institute for Brain ScienceSeattle, WA, USA

**Keywords:** masking, backward masking, interocular suppression, continuous flash suppression, face processing, priming, awareness, consciousness

## Abstract

Different combinations of forward and backward masking as well as interocular suppression have been used extensively to render stimuli invisible and to study those aspects of visual stimuli that are processed in the absence of conscious experience. Although the two techniques—masking vs. interocular suppression—obviously differ both in their applications and mechanisms, only little effort has been made to compare them systematically. Yet, such a comparison is crucial: existing discrepancies in the extent of unconscious processing inferred from these two techniques must be reconciled, as our understanding of unconscious vision should be independent of the technique used to prevent visibility. Here, we studied similarities and differences between faces rendered invisible by masking vs. interocular suppression using a priming paradigm. By carefully equating stimulus strength across the two techniques, we analyzed the effects of face primes with the same viewpoint (repetition priming, Experiment 1) and of face primes with a different viewpoint (identity priming, Experiment 2) on the reaction times for a fame categorization task. Overall, we found that the magnitude of both repetition and identity priming largely depended on stimulus visibility. Moreover, when the primes were subjectively invisible, both repetition and identity priming were found to be qualitatively stronger under masking than under interocular suppression. Taken together, these results help refine our understanding of which level of visual processing each technique disrupts, and illustrate the importance of systematic methodological comparisons in the field of unconscious vision.

## Introduction

In pursuing the neural correlates of consciousness, neuroscientists have developed a number of experimental techniques for suppressing conscious awareness of visual stimuli while still allowing some degree of unconscious processing (for review see Kim and Blake, 2005).

Arguably, two of the most used techniques employed to study unconscious vision are a combination of forward and backward masking and interocular suppression. In forward and backward masking, a high-contrast mask image is shown respectively before and after a briefly presented prime stimulus, rendering the prime undetectable (Breitmeyer and Ogmen, 2006). In interocular suppression (IS), an image in one eye is suppressed via the presentation of a high-contrast mask in the opposite eye (Tong et al., 2006; Lin and He, 2009); continuous flash suppression (CFS) extends this technique by updating the mask several times per second, which allows for long-lasting and powerful image suppression (Tsuchiya and Koch, 2005). It is not always clear why a researcher chooses one technique over the other to conduct a specific experiment, as justification is typically not required. One key determinant is the desired suppression time: whereas a combination of forward and backward masking (sometimes referred to as sandwich masking, but for the rest of the article for simplicity referred to as masking—abbreviated M) is mostly appropriate for brief presentation of unconscious stimuli, CFS allows longer unconscious stimulation (note that variations on masking may be used for longer suppression periods, see Macknik and Livingstone, 1998). Given that CFS relies on binocular interactions while masking does not, it is likely that these two techniques achieve subjective invisibility in fundamentally different ways.

A meta-analysis would unfortunately fall short of making strong claims about a possible difference in the depth of unconscious processing between the two techniques. Given the unavoidable idiosyncrasies of each published study, too many uncontrolled variables could account for any differences that would be found in a meta-analysis. Some authors have recently attempted to compare these two suppression techniques empirically. Kanai et al. (2010) measured the confidence in reporting the absence of a stimulus rendered invisible by several techniques including masking and IS; they found that both masking and IS disrupted stimulus visibility by reducing the strength of sensory (input) signals, but other techniques such as the attentional blink disrupted attentional access to the sensory signals instead. Focusing on the processing of emotional faces, Stein et al. (2013) showed that visibility of emotional faces was mostly driven by high spatial frequencies both under masking and IS. Hence within the scope of these two studies, no difference was found between how masking and IS prevent visibility. Yet, the two techniques obviously find their origins in distinct mechanisms: for one thing, masking is monocular while IS is binocular (see Breitmeyer and Ogmen, 2006 for review). One may thus expect to find differences in the depth of processing allowed by each of the two techniques. In an influential study, Almeida and colleagues found that masking, but not IS, allowed for unconscious processing of non-manipulable objects (i.e., animal pictures) along the ventral visual pathways (Almeida et al., 2008). Looking at the processing of emotional faces, Faivre et al. (2012) failed to find a difference between masking and IS: in their hands, only low-level facial features were processed when emotional faces were rendered invisible by masking or IS (they also found that more complex features such as those encoding happy facial expressions were processed in similar conditions of invisibility in a crowding paradigm). Almeida et al. (2013) challenged this result: they reported that features conveying the expression of happiness and anger were indeed processed when rendered invisible by masking, but only the features conveying anger were processed under IS. The authors suggested a dissociation between a subcortical route involved in the processing of anger (available under both masking and IS), and a cortical route for the processing of happiness (available under masking only). Though these published studies report conflicting results, they illustrate an increasing concern regarding the possibility that unconscious processes may differ under different suppression techniques.

Not only is it necessary to compare two techniques within the same study (as in the few studies that we briefly reviewed above), it is also crucial to carefully equate as many parameters as possible between the techniques under scrutiny. Here, we sought to examine the differences in unconscious processing under masking and IS while matching stimulation conditions to the best of our ability. We chose a fame categorization task with a priming paradigm, building on previous results in the literature (Henson et al., 2008; Kouider et al., 2009). Priming effects have been used extensively as a measure of unconscious processing: they quantify whether the presence of an invisible stimulus facilitates the processing of a target stimulus sharing some similarities with that invisible prime (Kiesel et al., 2007). By varying the type of information shared between the prime and target, one can infer the level of processing undergone by the prime (e.g., from low-level featural information like orientation or color, to high-level information like semantic or emotional content). In this study, we chose to focus on the processing of face identity. There is compelling evidence for face identity processing under masking (see Kouider and Dehaene, 2007 for review); however, several studies suggest that the processing of face identity is disrupted under IS (see Faivre et al., 2014, in this volume for a review). Notably, identity after-effects (i.e., a bias for the perception of a specific face after the observer adapts to a face that has opposite global features) vanished when the face adaptor was rendered invisible by interocular suppression (Moradi et al., 2005). We looked into this apparent difference in unconscious processing depth between the two techniques using both repetition and identity priming effects for both famous and unfamiliar faces, with the hypothesis that we would replicate previous repetition and identity priming effects under masking, but find only repetition priming under IS. We randomly used masking or IS on each trial to render the primes invisible; the stimuli were carefully designed in such a way that participants did not notice this manipulation. We investigated two levels of face identity representation: viewpoint-dependent (i.e., repetition priming, Experiment 1) and viewpoint-independent (i.e., identity priming, Experiment 2). By varying the mask contrast used in masking and IS, we looked at repetition and identity priming effects as a function of stimulus visibility under each technique. While we did not find definite evidence supporting our specific prediction, we discovered variations in effect sizes between masking and IS indicating subtle differences between the two techniques.

## Materials and methods

### Participants

Forty four subjects participated in the study—18 (7 male, 11 female) for an experiment utilizing same-view priming (Experiment 1), and 26 (11 male, 15 female) for an experiment utilizing different-view priming (Experiment 2). All subjects were between 20 and 35 of age, reported normal or corrected-to-normal vision, and gave written statements of informed consent to participate in the study. All experiments conformed to Institutional Guidelines and to the Declaration of Helsinki.

### Apparatus

Stimuli were displayed on a Mitsubishi Diamond pro 2070 1024 × 768 px CRT monitor with a 100 Hz refresh rate. Subjects viewed the stimuli from a distance of 40 cm, through a set of mirrors, such that the left eye saw the left half of the screen, and the right eye saw the right half of the screen. The experiment was written and executed using Matlab and Psychophysics toolbox version 3.1 (Brainard, 1997; Pelli, 1997; Kleiner et al., 2007). Statistical analysis was performed using Matlab and R (R Foundation for Statistical Computing Vienna, Austria).

### Stimuli

**Figure 1:**
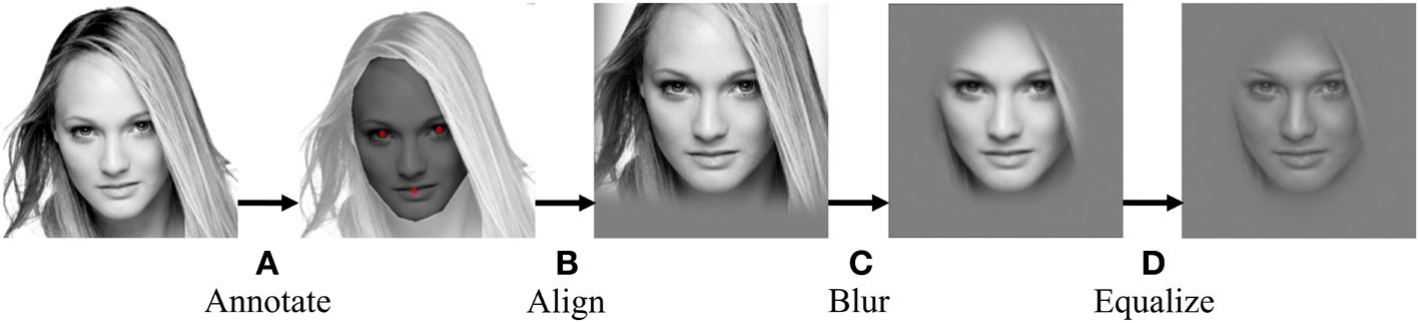
**The image normalization procedure, as executed on an unfamiliar female face. (A)** The experimentalist supplies annotation of mouth and eye locations. **(B)** These are used to center and scale the face to a standard shared across images; resulting visible image borders are blurred to gray. **(C)** A tight aperture is applied to the image. **(D)** The SHINE toolbox for MATLAB (Willenbockel et al., 2010) is used to normalize image histograms over the entire dataset.

The set of famous faces comprised 31 females and 30 males, all famous actors or politicians within the United States (for names, see Supplementary Table 1). The set of unfamiliar faces comprised 10 females and 10 males; they were, in fact, pictures of Israeli celebrities that were chosen to ensure rough equivalence in attractiveness and image quality with the famous faces. All face images used in the experiment were processed to remove most low level differences: they were converted to gray scale then normalized using a combination of in-house Matlab code and functions from the SHINE toolbox (Willenbockel et al., 2010 and Figure 1). Raw face images were 400 × 400 px in size. Our normalization procedure included the following steps: image transformation to match face sizes and positions based on manually annotated eye and mouth points (using Procrustes analysis, i.e., translation, rotation and global scaling); application of a Gaussian aperture and blur to remove image background and borders; and image histogram equalization over the entire set of face images. Histogram equalization was performed only on manually annotated face regions in each image, such that background color remained uniform across all stimuli. Targets and masks were finally scaled to occupy a visual angle of 13.5°, and primes 11.1°. The masks consisted of randomly generated white, black, and gray filled ovals superimposed onto each other (the dimensions of the oval shapes ranged between 4 and 22% of the size of the masks; 1000 oval shapes were randomly generated and pasted sequentially at random locations within each mask); we generated a pool of 1000 such masks, which we sampled from randomly, without replacement, in each trial.

### Procedure

Upon arrival, participants were asked to look over the set of famous male and female faces, from which they picked a subset of 10 of each gender that they were most familiar with. In Experiment 1, subjects were exposed to frontal views of each face; in Experiment 2, subjects were exposed to both frontal and profile views of each face, and were told to only choose faces which they could recognize from both points of view. Subjects who did not feel sufficiently familiar with the faces to accomplish the task were not tested further. Only one subject was rejected at this stage. No subject reported familiarity with any the 20 unfamiliar faces.

**Figure 2:**
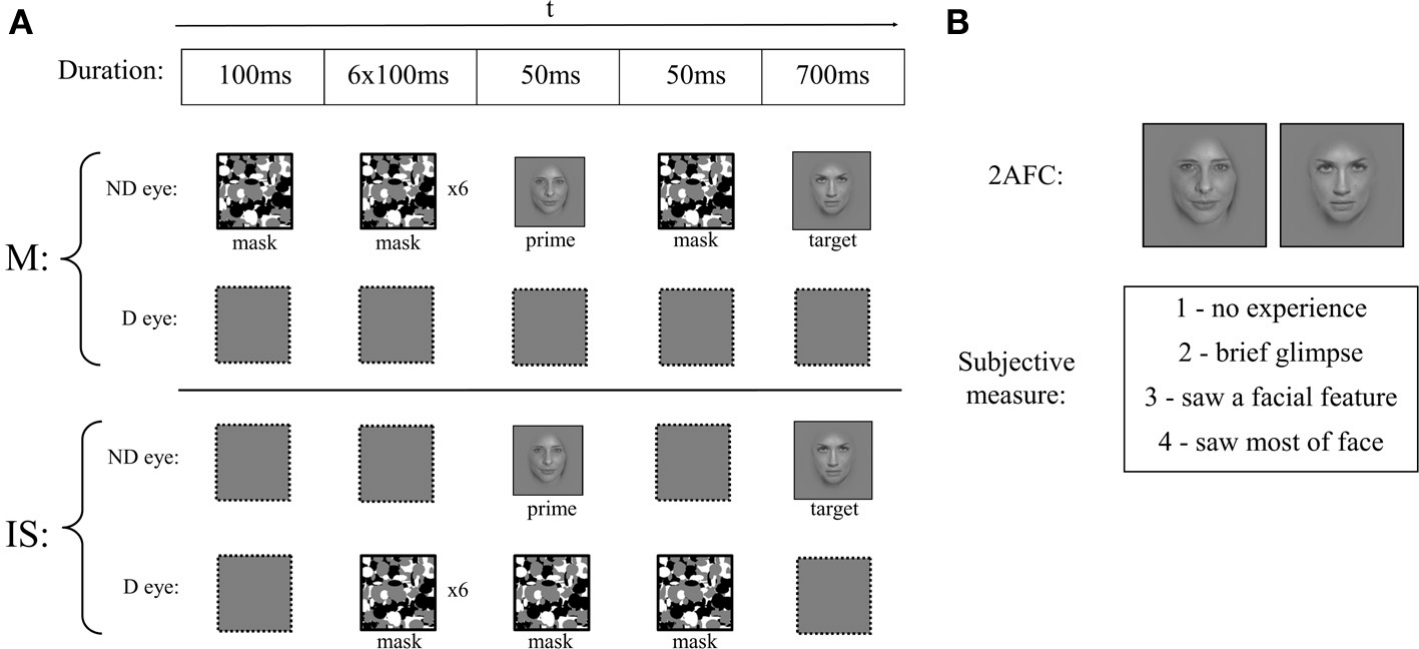
**A schematic depiction of a single trial that required subjects to make 3 responses. (A)** Each trial was displayed with either masking (M) or interocular suppression (IS) applied to the prime. The sequence of images presented to the subjects' non-dominant (ND) and dominant (D) eye is shown. In masking, which is a combination of forward and backward masking, nothing is presented to the D eye, while under interocular suppression, masks are presented to the D eye and primes and targets are presented to the ND eye. Subjects had to categorize the target as famous or unfamiliar as quickly as possible using two arrow keys. **(B)** After categorization, they were asked to indicate which face was presented as a prime among two alternatives (2-AFC objective visibility measure) and, immediately afterwards, to indicate the subjective experience they had about the prime on a 4-point Perceptual Awareness Scale (subjective visibility measure).

Each trial began with a fixation cross that stayed on the screen until a button press (Figure 2). The subjects' main task was to categorize a target image as famous or unfamiliar as fast as possible using the arrow keys (using the right and left arrow keys on the keyboard respectively, with the ring and index fingers of the right hand). The masking technique in each trial was randomly chosen as either masking or interocular suppression (IS). In masking trials, eight different masks (to match the sequence of mask in interocular suppression) were presented for 100 ms each, followed by a 50 ms prime, a 50 ms mask, and finally a 700 ms target image; all masks, the prime, and the target image were presented to the subject's non-dominant eye, while isoluminant gray was presented to the dominant eye. In IS trials, all masks were presented to the dominant eye, while the prime and the target image were presented to the non-dominant eye. The sequence and timing of masks, prime, and target image was the same as in masking, with the exception that the first mask in the eight-mask sequence was removed to counterbalance the addition of a mask shown simultaneously with the prime (necessary to induce interocular suppression). There was no enforced time-out; however if subjects took longer than 1.2 s on the speeded target categorization task, they were presented with a penalizing “Too Slow!” message for 2 s. Two questions aimed at assessing trial-by-trial visibility followed the target categorization task. First, a two-alternative forced choice task (2-AFC) in which two faces were shown side-by-side and subjects had to pick which of the two was shown as the prime face. The choices were always faces from the same viewpoint as the prime (see Experimental Design below). The target image (or, in Experiment 2, the same identity as the target image, seen from a different viewpoint) was always one of the choices, with the other image being chosen to ensure that the prime was always available as a choice (i.e., if prime and target were the same, the second image would be a random other image with the same fame; otherwise, the other image would be the prime). This 2-AFC served as an objective measure of visibility. Second, subjects rated their subjective experience of the prime using the following options “1—no experience”; “2—brief glimpse”; “3—saw a facial feature”; and “4—saw most of face.” Importantly, they were instructed that “2—brief glimpse” meant they detected some shapeless luminance blob in which they did not detect any facial features (as opposed to “3—saw a facial feature”). This served as a subjective measure of visibility (Ramsøy and Overgaard, 2004). Note that subjects always performed a practice version of the task for several minutes to ensure that they understood instructions properly.

### Experimental design

The relationship between the target and the prime was varied in a controlled manner across trials. Primes and targets were always gender and fame matched, to minimize response congruity effects (Damien, 2001; Kouider et al., 2009). In trials of the same-view priming experiment (Experiment 1), both the prime and the target were drawn from a set of front-view-only faces. Priming relationship could thus either be “same view, different identity (same fame)” (e.g., a front-view face X as a prime, followed by another front-view face Y as a target, both being either famous or unfamiliar) or “same view, same identity” (e.g., a front-view face X as a prime, followed by the same front-view face X as a target). In trials of the different-view priming experiment (Experiment 2), the targets were drawn from the same set of front-view-only faces as in Experiment 1, while the primes were drawn from a set of corresponding quarter-profile-view faces. Priming relationships could thus be “different view, different identity (same fame)” (e.g., quarter-profile-view face X as a prime, followed by the front-view face Y as a target, both being either famous or unfamiliar) or “different view, same identity” (e.g., quarter-profile-view face X as a prime, followed by the front-view face X as a target). To minimize priming across trials (for example, if target face X is presented on trials *n-1* and *n*, the response on trial *n* is likely to be sped up), we ensured that a given famous or unfamiliar face was never seen in consecutive trials as either the prime, the target, or the alternate choice in the 2-AFC question. Note that primes were scaled to be 80% the size of targets in order to minimize pixel overlap in Experiment 1, as has been done in previous studies (Kouider et al., 2009). For consistency, primes were also scaled to 80% in Experiment 2 (even though pixel overlap was not a concern). We had three levels of masks contrast to vary masking strength across trials: 2, 40, and 60% (Michelson contrast). Pilot experiments suggested that these values were associated respectively to conditions of full visibility, partial visibility, and null visibility of the primes. Both experiments were broken up in blocks of 144 trials, each containing six repetitions of each condition; subjects completed as many blocks (up to five) as they could within 90 min.

### Analysis

Five subjects (three in Experiment 1, two in Experiment 2) with below 70% accuracy on the target categorization task were excluded from analysis (their low accuracy indicates that they were not familiar enough with the famous faces; also, since all trials for which target categorization was incorrect are removed from the analysis, the number of trials for these subjects becomes too low for accurate estimates of priming effects). Across the remaining subjects, the first 10 trials were discarded to allow subjects to reach a stable strategy, as were trials in which they categorized the target incorrectly (as mentioned above), too quickly compared to a hard threshold (reaction times less than 200 ms were excluded), or too quickly or slowly as measured by a deviation of more than two standard deviations from each subject's mean reaction time. After these restrictions, if for any subject the number of trials for a given mask contrast, target fame, masking technique and prime-target relationship was less than or equal to five, all trials for that subject at that mask contrast were discarded (all trials at that mask contrast were discarded, instead of just trials within the specific combination of mask contrast, target fame, masking technique and prime-target relationship, to maintain a balanced design for ANOVAs within each mask contrast). In order to satisfy assumptions of data normality, we performed statistical tests on the inverse of reaction times (Whelan, 2008).

Transformed reaction times were analyzed with 3 × 2 × 2 × 2 repeated measures ANOVA, with mask contrast, masking technique, target fame, and prime-target relationship as within subject factors, and subjects as a random variable. Priming effects were calculated by subtracting mean reaction times in the unrelated (different identity) vs. related (same identity) conditions. Therefore, positive priming values reflect a decrease of reaction times in related vs. unrelated trials. Where significant interactions arose, planned *t*-tests were performed. Similar ANOVAs run on accuracies in the target categorization task did not yield any significant effects. Finally, similar ANOVAs were run on accuracies in the objective visibility task in order to estimate the visibility of the primes. No correction for multiple comparisons was performed.

## Results

The number of trials for each subject after the eliminations described in Materials and Methods are listed in Supplementary Tables 2, 3. Qualitative Q-Q plots demonstrating the utility of the inverse transform in upholding the assumption of normality are shown in Supplementary Figures 1–4. No subject independently reported being able to differentiate the different masking techniques.

### Experiment 1

**Figure 3:**
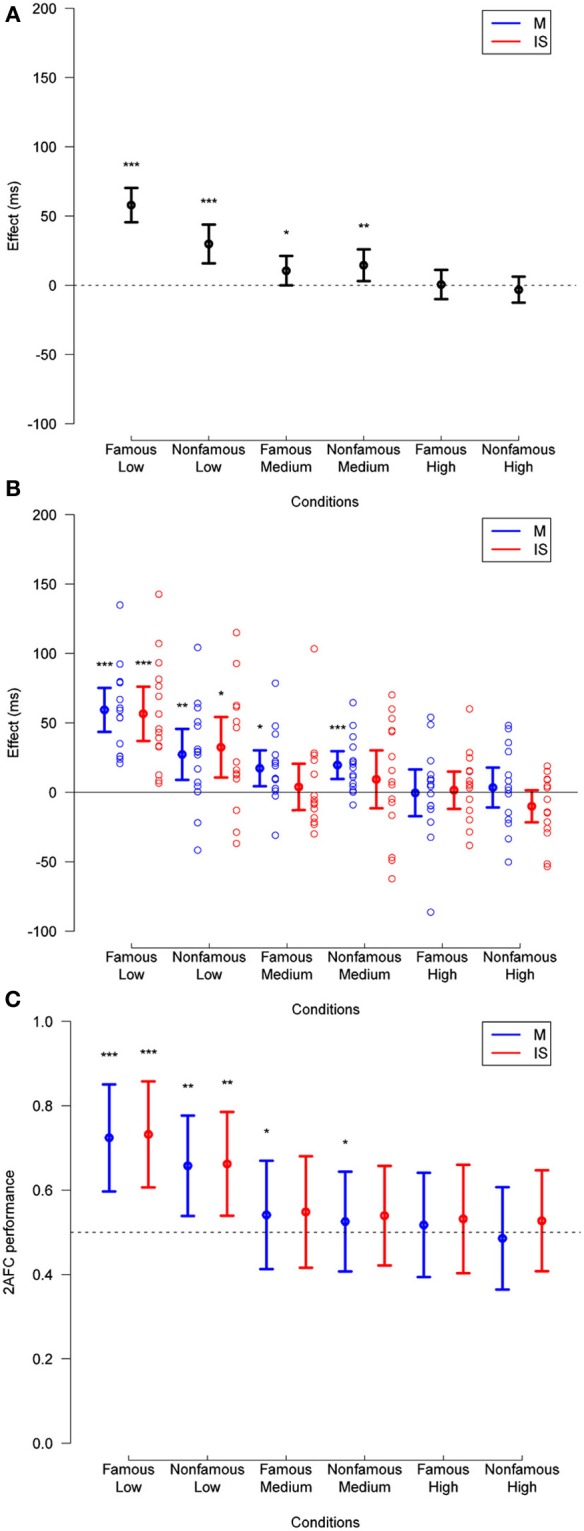
**(A)** Priming effect observed in Experiment 1, across fame of target and prime (famous or unfamiliar), and mask contrast levels (low, medium, or high), for trials with any subjective visibility rating. **(B)** Same data broken down by masking method. The data for individual subjects is shown (empty circles). **(C)** Corresponding 2-AFC performance is collapsed across relation. In all plots, deviation of effects from a null hypothesis are measured in paired *t*-tests, and are not corrected. ^*^p < 0.05, ^**^p < 0.01, ^***^p < 0.001.

Priming effects are displayed in Table 1. We started by running a 3 × 2 × 2 × 2 repeated measures ANOVA across all trials without selection by subjective visibility rating (Figure 3; see Materials and Methods). It showed a main effect of relation [*F*_(1, 14)_ = 33.09, *p* < 0.001], indicating that participants categorized the fame of a target face faster when it was preceded by an identical then by a different prime face (i.e., repetition priming effect, mean reaction times difference = 18 ms, *SD* = 16 ms). The same analysis also showed a main effect of target fame [*F*_(1, 14)_ = 23.96, *p* < 0.001], and mask contrast [*F*_(2, 28)_ = 7.28, *p* = 0.003], indicating respectively that participants responded faster to famous than to unfamiliar faces (mean reaction times difference = 44 ms, *SD* = 36 ms), and to low than to high mask contrasts (weak contrast: 641 ms, *SD* = 64 ms; medium contrast: 651 ms, *SD* = 56 ms; high contrast: 656 ms, *SD* = 53 ms).

**Table 1:** **Reaction time priming effect sizes for Experiment 1**.

**Method**	**Fame**	**Contrast**	**All Subj. Vis**.		**Subj. Vis. 1-2**	
			**Effect (ms)**	***SD* (ms)**	**Effect (ms)**	***SD* (ms)**
M	Famous	1	59	31	–	–
IS	Famous	1	57	39	–	–
M	Unfamiliar	1	27	36	–	–
IS	Unfamiliar	1	32	43	–	–
M	Famous	2	17	25	14	32
IS	Famous	2	4	33	15	41
M	Unfamiliar	2	20	20	18	24
IS	Unfamiliar	2	9	41	6	42
M	Famous	3	0	33	−7	38
IS	Famous	3	2	27	6	24
M	Unfamiliar	3	4	29	5	35
IS	Unfamiliar	3	−10	23	−9	25

The ANOVA also showed an interaction between relation and target fame [*F*_(1, 14)_ = 11.28, *p* = 0.005], between relation and mask contrast [*F*_(2, 28)_ = 42.82, *p* < 0.001], and between target fame and mask contrast [*F*_(2, 28)_ = 4.72, *p* = 0.017]. These interactions respectively revealed that priming effects were larger for famous than for unfamiliar faces (mean priming difference = 9 ms, *SD* = 20 ms), decreased as mask contrast increased (weak contrast: 44 ms, *SD* = 29 ms; medium contrast: 13 ms, *SD* = 15 ms; high contrast: −1 ms, *SD* = 16 ms), and that mask contrast affected reaction times more for famous faces (weak contrast: 613 ms, *SD* = 52 ms; medium contrast: 631 ms, *SD* = 50 ms; high contrast: 637 ms, *SD* = 53 ms) than for unfamiliar faces (weak contrast: 668 ms, *SD* = 80 ms; medium contrast: 672 ms, *SD* = 69 ms; high contrast: 674 ms, *SD* = 58 ms) (though this last interaction is unrelated to the priming effects, we report it here for completeness).

Finally, we found a triple interaction between relation, mask contrast, and target fame [*F*_(2, 28)_ = 8.92, *p* = 0.001], suggesting that the magnitude of priming effects decreased more as mask contrast increased for famous than for unfamiliar faces. No other effect reached significance (*p*-values > 0.28).

Importantly, no effect of technique (i.e., masking vs. interocular suppression) reached significance (*p* > 0.28). That is, the magnitude of differences in reaction times was not affected by which one of the two techniques rendered the prime invisible.

Regarding stimulus visibility, a 3 × 2 × 2 × 2 repeated measures ANOVA across the same trials showed main effects of target fame [*F*_(1, 14)_ = 10.84, *p* = 0.005] and mask contrast [*F*_(1, 14)_ = 13.16, *p* < 0.001]. That is, accuracy on the objective visibility task was higher for famous (mean = 0.60, *SD* = 0.09) than for unfamiliar faces (mean = 0.57, *SD* = 0.07) and it decreased as mask contrast increased (weak contrast: 0.69, *SD* = 0.19; medium contrast: 0.54, *SD* = 0.058; high contrast: 0.52, *SD* = 0.048). The ANOVA also revealed an interaction between target fame and mask contrast [*F*_(2, 28)_ = 4.27, *p* = 0.024]; that is, the strength of masking had a higher impact on famous than on unfamiliar faces (mean of the accuracy difference, i.e., famous accuracy minus unfamiliar accuracy; at weak mask contrast: 0.068, *SD* = 0.075; medium mask contrast: 0.012, *SD* = 0.050; high mask contrast: 0.018, *SD* = 0.055). No other effect reached significance (*p*-values > 0.21).

Taken together, as the ANOVA showed an impact of mask contrast on priming independently of the technique (i.e., no effect of technique reached significance), the results suggest masking and IS have a similar detrimental effect on the magnitude of repetition priming.

**Figure 4:**
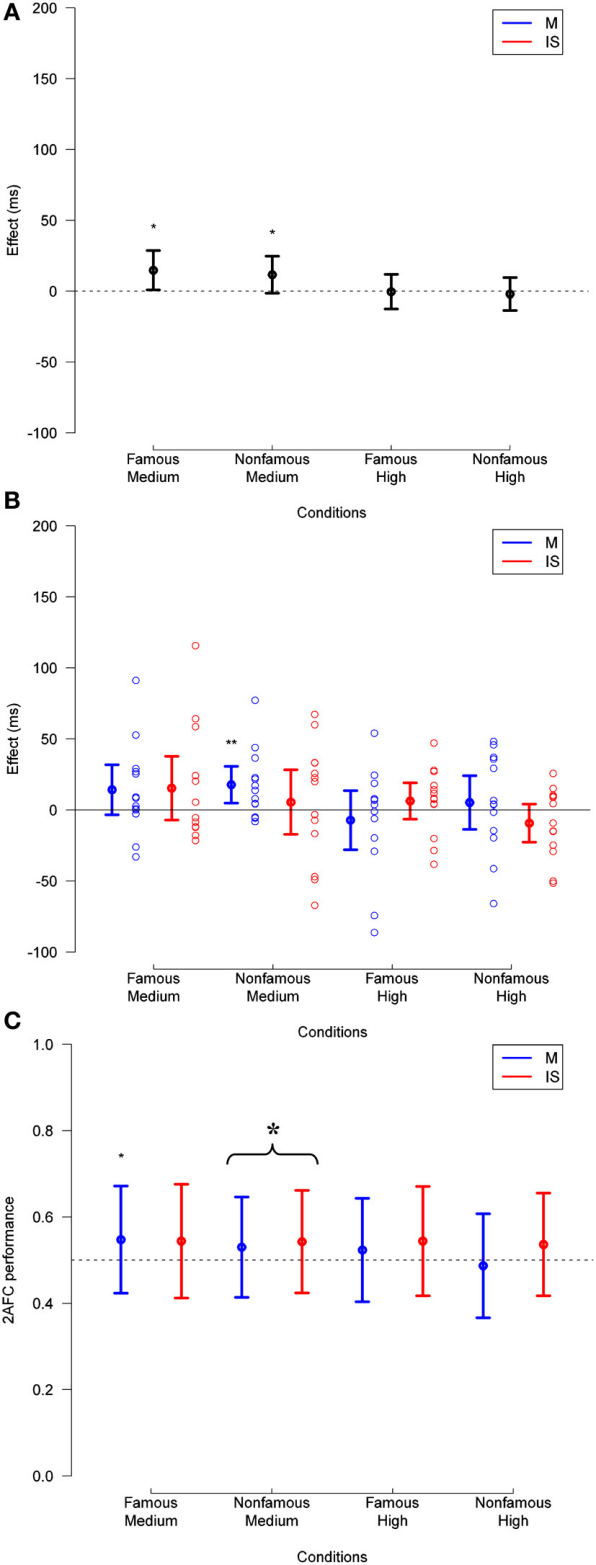
**As in Figure 3 except for only those trials in which the visibility was 1 or 2**. Deviation of 2-AFC performance from chance when collapsed across method (as reported in Results) is also displayed in **(C)**. See legend to Figure 3 for other details.

In order to test for the existence of unconscious repetition priming under masking and IS, we repeated the same analysis but only on those trials in which participants either reported “no experience,” or “a brief glimpse” of the prime (Figure 4; see Materials and Methods). With additional elimination due to a decreased number of trials in each condition (see Materials and Methods), this reduced total trials by 26.1% relative to the previous analysis. Subjective visibility ratings, averaged across subjects, mask contrast, target fame, and masking technique, are presented in Supplementary Figure 5. This analysis included only medium and high mask contrast, as only 6 subjects fulfilled the selection criteria (see Materials and Methods) in the weak mask contrast condition (this is, of course, expected: the weak mask contrast lead to mostly visible trials).

This analysis showed a main effect of target fame [*F*_(1, 12)_ = 13.90, *p* = 0.003], and mask contrast [*F*_(1, 12)_ = 5.83, *p* = 0.03], revealing that participants responded faster to famous faces than to unfamiliar faces (mean reaction times difference = 36 ms, *SD* = 36 ms), and to medium than to high mask contrasts (mean reaction times difference = 5 ms, *SD* = 11 ms). Here, the main effect of relation was not significant [*F*_(1, 12)_ = 4.42, *p* = 0.055; mean reaction times difference = 6 ms, *SD* = 14 ms]; that is, we could not reject the hypothesis of no repetition priming, over all conditions. The ANOVA did show an interaction between relation and mask contrast [*F*_(1, 12)_ = 7.1, *p* = 0.02], revealing that priming effects decreased as mask contrast increased (at medium mask contrast: 13 ms, *SD* = 15 ms, *t*_(12)_ = 3.11, *p* = 0.009; at high mask contrast: −1 ms, *SD* = 19 ms, *t*_(12)_ = −0.23, *p* = 0.82). Again, no effect of masking technique reached significance (*p*-values > 0.19).

Regarding stimulus visibility, a 2 × 2 × 2 × 2 repeated measures ANOVA across the same trials showed an interaction between target fame and mask contrast [*F*_(1, 12)_ = 7.88, *p* = 0.02], revealing that the strength of masking had a higher impact on famous than on unfamiliar faces (mean of the accuracy difference at medium mask contrast: 0.0095, *SD* = 0.058; high mask contrast: 0.02, *SD* = 0.060). No other effect reached significance (*p*-values > 0.06).

In addition, *post-hoc* one-sample *t*-tests revealed that 2-AFC performance across mask strength differed from chance for unfamiliar faces [mean = 0.54, *t*_(12)_ = 2.54, *p* = 0.03], but did not differ significantly for famous faces [mean = 0.55, *t*_(12)_ = 1.93, *p* = 0.08]. For the high mask contrast, 2-AFC performance was indistinguishable from chance for both famous [mean = 0.53, *t*_(12)_ = 2.11, *p* = 0.06] and unfamiliar faces [mean = 0.51, *t*_(12)_ = 0.75, *p* = 0.50].

At medium mask contrast, repetition priming was significant for masking [16 ms, *t*_(12)_ = 3.54, *p* = 0.004], but not under IS [10 ms, *t*_(12)_ = 1.71, *p* = 0.11]. Separating by the fame of the target, repetition priming remains significant for masking for unfamiliar faces [18 ms, *t*_(12)_ = 3.14, *p* = 0.009], but loses significance for famous faces [14 ms, *t*_(12)_ = 1.91, *p* = 0.081], and remains non-significant for IS for both famous [15 ms, *t*_(12)_ = 1.39, *p* = 0.19] and unfamiliar [6 ms, *t*_(12)_ = 0.90, *p* = 0.39] faces. No repetition priming was found at high mask contrast (*p*-values > 0.32).

These results suggest that, if awareness is defined according to subjective visibility (subjects report seeing nothing, or a brief glimpse with no content), unconscious repetition priming occurs under masking at medium, but not high mask contrast. Priming appeared slightly more robust under masking than under IS, but no significant difference of priming depending on the technique reached significance in the ANOVA. Note that if we define awareness with the objective 2-AFC performance measure, no claim of unconscious priming can be made because subjects were on average slightly above chance (Figures 3C, 4C). We come back to these issues in the discussion.

### Experiment 2

**Figure 5:**
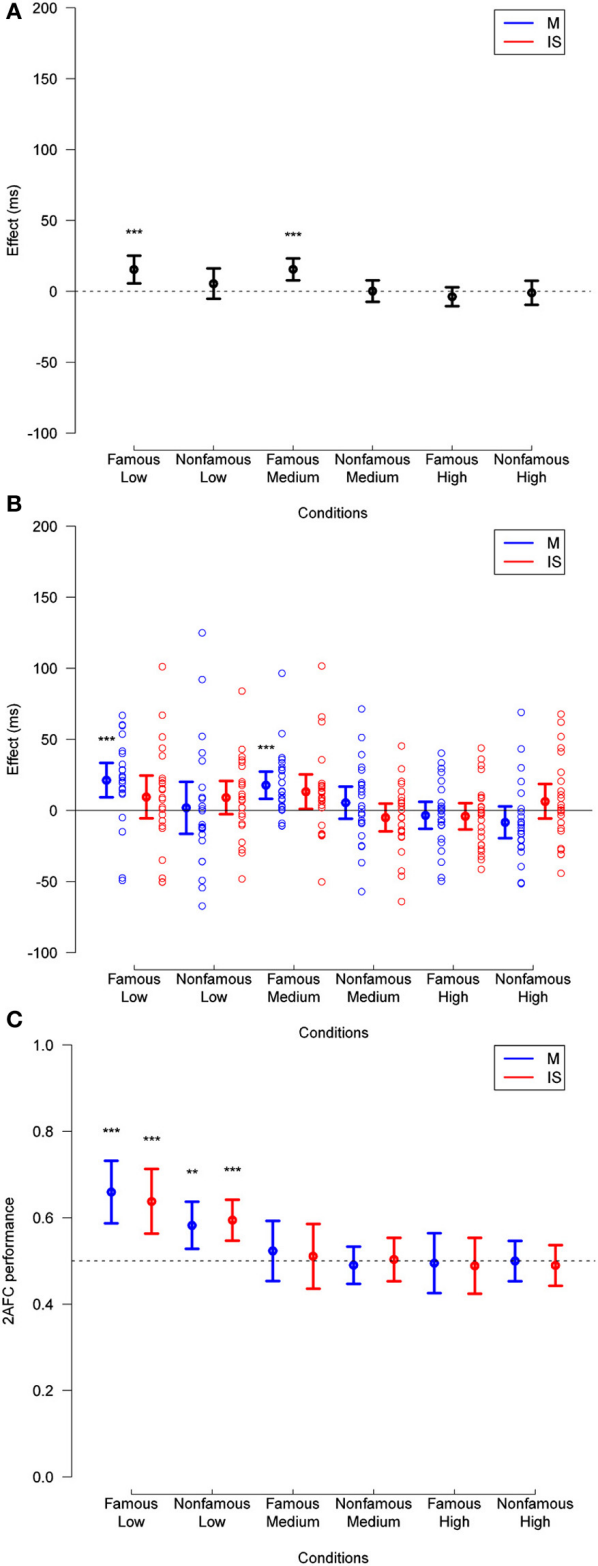
**(A)** Priming effect observed in Experiment 2, across fame of target and prime (famous or unfamiliar), and mask contrast levels (low, medium, or high), for trials with any subjective visibility rating. **(B)** Same data broken down by masking method. The data for individual subjects is shown (empty circles). **(C)** Corresponding 2-AFC performance is presented collapsed across relation. In all plots, deviation of effects from a null hypothesis are measured in paired *t*-tests, and are not corrected. ^*^p < 0.05, ^**^p < 0.01, ^***^p < 0.001.

Priming effects are displayed in Table 2. We tested for viewpoint-independent priming in this experiment by running a 3 × 2 × 2 × 2 repeated measures ANOVA across all trials without selection by subjective visibility rating (see Materials and Methods and Figure 5). It showed a main effect of relation [*F*_(1, 22)_ = 9.48, *p* = 0.006], target fame [*F*_(1, 22)_ = 33.60, *p* < 0.001], and mask contrast [*F*_(2, 45)_ = 7.50, *p* = 0.002]. As in Experiment 1, these effects were respectively due to participants responding faster in related than in unrelated trials (i.e., priming effect: mean reaction times difference = 6 ms, *SD* = 9 ms), famous vs. unfamiliar faces (mean reaction times difference = 44 ms, *SD* = 37 ms), and low vs. high mask contrasts (weak contrast: 661 ms, *SD* = 67 ms; medium contrast: 669 ms, *SD* = 64 ms; high contrast: 675 ms, *SD* = 66 ms).

**Table 2:** **Reaction time priming effect sizes for Experiment 2**.

**Method**	**Fame**	**Contrast**	**All Subj. Vis**.		**Subj. Vis. 1-2**	
			**Effect (ms)**	**SD (ms)**	**Effect (ms)**	**SD (ms)**
M	Famous	1	21	30	15	33
IS	Famous	1	9	37	15	44
M	Unfamiliar	1	2	45	1	57
IS	Unfamiliar	1	9	29	9	34
M	Famous	2	18	24	14	24
IS	Famous	2	13	30	13	29
M	Unfamiliar	2	6	28	4	32
IS	Unfamiliar	2	−5	25	−7	24
M	Famous	3	−3	24	−5	28
IS	Famous	3	−4	23	−2	28
M	Unfamiliar	3	−8	28	−11	31
IS	Unfamiliar	3	6	30	5	33

The ANOVA also showed an interaction between relation and target fame [*F*_(1, 22)_ = 8.90, *p* = 0.007], between relation and mask contrast [*F*_(2, 45)_ = 8.68, *p* < 0.001]. These interactions respectively revealed that priming effects were larger for famous than for unfamiliar faces (mean reaction times difference = 8 ms, *SD* = 18 ms), and that priming effects decreased as mask contrast increased (weak contrast: 10 ms, *SD* = 17 ms; medium contrast: 8 ms, *SD* = 14 ms; high contrast: −2 ms, *SD* = 14 ms).

**Figure 6:**
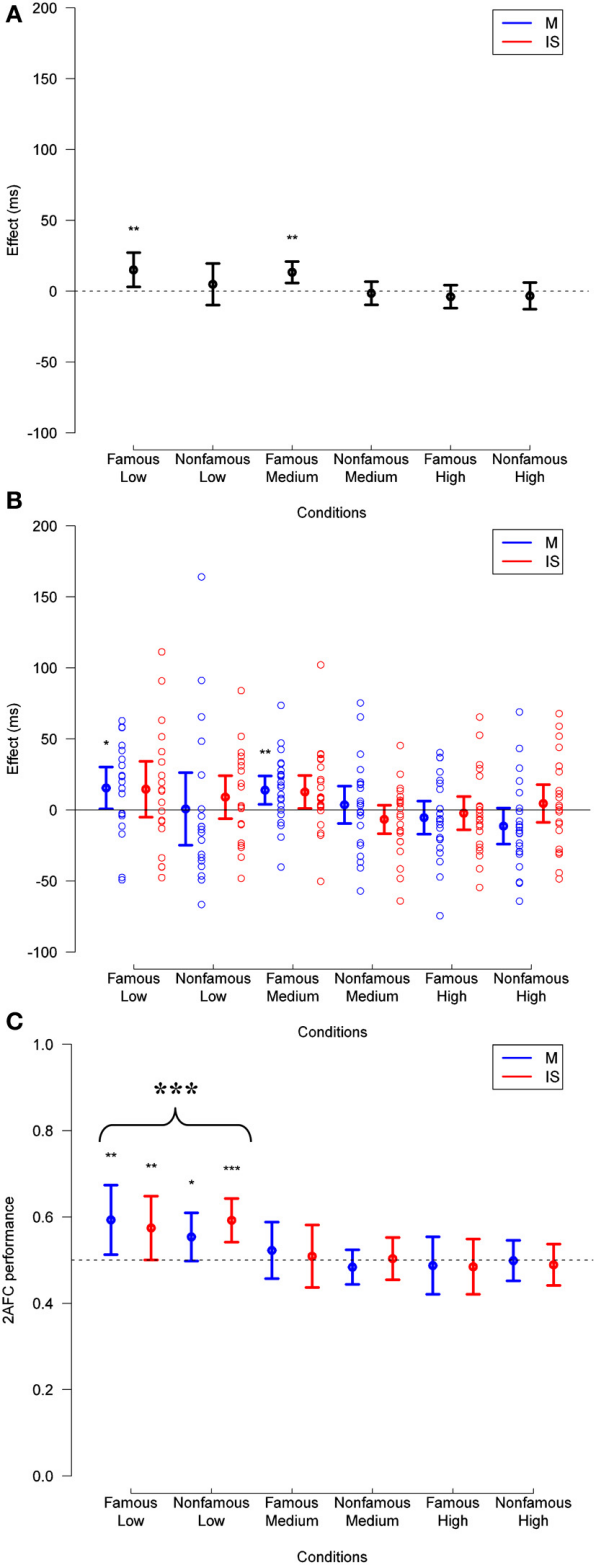
**As in Figure 5 except for only those trials in which the visibility was rated 1 or 2**. Deviation of 2-AFC performance from chance when collapsed across fame and method (as reported in Results) is also displayed in **(C)**. See legend to Figure 5 for other details.

Finally, we found a triple interaction between relation, mask contrast, and target fame [*F*_(2, 45)_ = 3.47, *p* = 0.040], suggesting that the magnitude of priming effects decreased more as mask contrast increased for famous than for unfamiliar faces (Figure 6), and a triple interaction between masking technique, mask contrast, and target fame [*F*_(2, 45)_ = 4.54, *p* = 0.016], revealing that the difference in reaction time between masking and IS depended more strongly on mask contrast for famous faces (weak contrast: 8 ms, *SD* = 18 ms; medium contrast: −3 ms, *SD* = 28 ms; high contrast: 12 ms, *SD* = 25 ms) than for unfamiliar faces (weak contrast: 1 ms, *SD* = 21 ms; medium contrast: 2 ms, *SD* = 29 ms; high contrast: −2 ms, *SD* = 26 ms) (this effect is somewhat irrelevant, but reported for completeness). No other effect reached significance (*p*-values > 0.10).

Regarding stimulus visibility, a 3 × 2 × 2 × 2 repeated measures ANOVA across the same trials showed main effects of target fame [*F*_(1, 22)_ = 7.28, *p* = 0.013] and mask contrast [*F*_(2, 45)_ = 25.66, *p* < 0.001], revealing that accuracy on the objective visibility task was higher for famous (mean = 0.55, *SD* = 0.059) than for unfamiliar faces (mean = 0.53, *SD* = 0.040), and that it decreased as mask contrast increased (weak contrast: 0.62, *SD* = 0.10; medium contrast: 0.51, *SD* = 0.050; high contrast: 0.49, *SD* = 0.037). The ANOVA also revealed an interaction between target fame and mask contrast [*F*_(2, 45)_ = 4.27, *p* = 0.010], revealing that the strength of masking had a higher impact on performance for famous than for unfamiliar faces (mean of the accuracy difference at weak mask contrast: 0.06, *SD* = 0.087; medium mask contrast: 0.02, *SD* = 0.056; high mask contrast: −0.0030, *SD* = 0.068). No other effect reached significance (*p*-values > 0.09).

In order to test for the existence of unconscious identity priming under masking and IS, we ran the same 3 × 2 × 2 × 2 repeated measures ANOVA on trials in which participants reported having “no experience,” or just seeing “a brief glimpse” of the prime (see Materials and Methods). This eliminated an additional 12.8% of the total trials relative to the previous analysis. Subjective visibility ratings, averaged across subject, mask contrast, target fame, and masking technique, are presented in Supplementary Figure 6. As opposed to Experiment 1, we obtained enough trials at weak mask contrast to include this condition in the following analysis (Figure 6).

This analysis showed a main effect of mask contrast [*F*_(2, 40)_ = 7.09, *p* = 0.002], and target fame [*F*_(1, 21)_ = 32.60, *p* < 0.001], revealing that reaction times were longer as mask contrast increased (weak: 663 ms, *SD* = 64 ms; medium: 672 ms, *SD* = 61 ms; high: 680 ms, *SD* = 63 ms), and shorter for famous than for unfamiliar faces (mean reaction times difference = 42 ms, *SD* = 38 ms). The main effect of relation was not significant [*F*_(1, 21)_ = 3.59, *p* = 0.072], suggesting that participants' response time in related vs. unrelated trials (i.e., priming effect, considered across all other conditions) was similar (mean reaction times difference = 3 ms, *SD* = 10 ms).

The ANOVA also showed an interaction between relation and mask contrast [*F*_(2, 40)_ = 5.98, *p* = 0.005], revealing that priming effects decreased as mask contrast increased. *Post-hoc t*-tests revealed that identity priming was significant at weak mask contrast [10 ms, *SD* = 19 ms, *t*_(18)_ = 2.77, *p* = 0.012] but not at medium mask contrast [6 ms, *SD* = 13 ms; *t*_(22)_ = 1.85; *p* = 0.078], nor at high mask contrast: [−4 ms, *SD* = 15 ms, *t*_(22)_ = −1.22, *p* = 0.24]. No other effects reached significance (*p*-values > 0.075).

Regarding stimulus visibility, a 3 × 2 × 2 × 2 repeated measures ANOVA across the same trials showed a main effect of mask contrast [*F*_(2, 40)_ = 12.84, *p* < 0.001]. Exploratory one-sample *t*-tests revealed that 2-AFC performance across a given mask strength differed from chance at the lowest mask contrast [mean = 0.58, *t*_(18)_ = 4.36, *p* < 0.001]. For the middle and high mask contrasts, 2-AFC performance was indistinguishable from chance [mean = 0.50, *t*_(22)_ = 0.45, *p* = 0.65 and mean = 0.49, *t*_(22)_ = −1.20, *p* = 0.24 respectively]. No other effect in the ANOVA reached significance (*p*-values > 0.13).

*Post-hoc t*-tests revealed that at medium mask contrast (i.e., the one at which unconscious processing is most likely to occur according to the visibility analysis described above), identity priming was significant for famous faces for masking [14 ms, *SD* = 5 ms, *t*_(22)_ = 3.22, *p* = 0.004], but not for IS [13 ms, *SD* = 6 ms, *t*_(22)_ = 1.71, *p* = 0.10]. Identity priming was not significant for unfamiliar faces under either masking [4 ms, *SD* = 7 ms, *t*_(22)_ = 0.18, *p* = 0.86] or IS [−7 ms, *SD* = 5 ms, *t*_(22)_ = −1.00, *p* = 0.33]. As opposed to what we found in Experiment 1, this suggests that unconscious identity priming is more robust for famous than for unfamiliar faces, and similarly to Experiment 1, for masking than for IS. However, a paired *t*-test between masking techniques at the medium mask contrast did not reveal a difference in famous identity priming [*t*_(22)_ = 0.53, *p* = 0.60], unfamiliar identity priming [*t*_(22)_ = 0.46, *p* = 0.65], or a collapsed condition [*t*_(22)_ = 0.61, *p* = 0.55].

## Discussion

In this work, we assessed the influence of a combination of forward and backward masking (referred to simply as “masking”) and interocular suppression (IS) on face processing. We took great care in carefully equalizing as many parameters as possible between the two techniques. This is the novel aspect of our study in contrast to previous studies that compared suppression techniques (Almeida et al., 2008, 2010, 2013; Kanai et al., 2010; Faivre et al., 2012; Stein et al., 2013); critically, the duration and energy of the masked stimulus was the same across the two suppression methods.

We respectively used repetition priming (Experiment 1) and identity priming (Experiment 2) as an index of low-level and high-level face processing. By manipulating mask contrast while keeping the prime contrast constant, we found that both masking and IS affected repetition and identity priming effects, as revealed by a decrease in priming magnitude when mask contrast increased. In both experiments, priming was virtually abolished at the highest mask contrast, which corresponded to chance-level performance in the objective visibility task. These results suggest that masking and IS already interfere before (or at the level of) low-level face processing as indexed by repetition priming. This is in line with previous results showing that the magnitude of tilt and motion after-effects (considered low-level effects) decreased when the strength of suppression by binocular rivalry and crowding increased (Blake et al., 2006).

Looking beyond this obvious general trend in our results, priming effects and prime visibility could be dissociated for masking at medium mask contrast. In Experiment 1, famous and unfamiliar faces induced repetition priming when considering only trials in which subjects reported not seeing the primes (note however that performance in the objective visibility task differed slightly from chance level). In Experiment 2, selecting the low visibility trials, only famous faces induced identity priming (and in that experiment, the objective measure of visibility was at chance). The fact that unfamiliar faces elicited repetition but not identity priming is consistent with the idea that unconscious repetition priming stems from low-level processes (possibly at the level of primary visual cortex, see Faivre and Koch, 2014), while unconscious identity priming stems from preexisting representations that are (re)activated by famous faces only (Henson et al., 2000). Interestingly, priming effects and visibility were not found to be significantly dissociated when prime stimuli were rendered invisible by IS. Beyond a mere absence of evidence, this negative finding could be meaningful, as stimulation conditions were perfectly equated between masking and IS (see Materials and Methods). This is notably corroborated by the fact that no observer independently reported the presence of two masking techniques.

A classical pitfall when arguing for the existence of unconscious effects is the possibility that the direct measure (here, objective visibility) is less sensitive than the indirect one (here priming). If this were the case in our experiment (due to unaccounted-for differences in task difficulty, or memory) priming could mistakenly be attributed to unconscious processes in conditions where objective visibility does not deviate from chance, while actually arising from isolated trials in which the prime stimulus was at least partly visible. We anticipated this issue by combining objective and subjective visibility measures on a trial-by-trial basis. Therefore, we could restrict the analysis of priming effects to those trials in which participants reported having no experience or just a brief glimpse of the prime face. Importantly, observers were asked to report seeing a “brief glimpse” only when they perceived meaningless luminance or contrast patterns (i.e., no facial features). When considering such trials only, performance in the objective visibility task dropped in all mask contrast conditions, although it still deviated significantly from chance in the medium mask contrast condition in Experiment 1, and at the weak mask contrast condition in Experiment 2. Two alternative hypotheses explain this surprising result. The first one is that some subjects performed the subjective visibility task incorrectly, erroneously reporting no experience of the prime's features while consciously seeing some of them. The second one is that subjects genuinely had no experience of the prime's features, but related unconscious representations lead them to still perform higher than chance-level in the objective visibility task. We cannot disentangle these two possibilities. Subjective measures of awareness (like the Perceptual Awareness Scale used here) can be seen as a more sensitive assessment of awareness than objective measures, such as the 2-AFC reported here (Cheesman and Merikle, 1986; Sandberg et al., 2010; see also Sandberg et al. in the current issue for a review of different visibility measures).

Taken together, our results echo, but do not entirely match previous results showing repetition and identity priming for famous but not for unfamiliar faces (Henson et al., 2008; Kouider et al., 2009). Note that in these last two studies, priming effects were possibly driven by residual visibility, as no trial-by-trial subjective visibility measure was performed [the authors relied instead on linear regression analyses as advocated by Greenwald et al. (1995) to claim unconscious priming, see Figure 2 in Henson et al., 2008 and Figure 1 in Kouider et al., 2009]. Regardless, our findings support the conclusion that the processing of facial identity (for faces previously known to the subject) can occur when stimulus awareness is prevented by masking.

The absence of identity priming under IS is in line with several studies that failed to demonstrate high-level face processing under IS at the behavioral level (see Faivre et al., 2014, in the current issue for a review). Although not reaching significance in the Null Hypothesis Significance Testing (NHST) framework commonly used in psychology (see recent discussions in the literature on replicability and the pitfalls of NHST, e.g., Cumming, 2013), one cannot help but notice that the mean priming effects we measured for the middle mask contrast under IS are positive; however, they show much greater inter-subject variability than in masking. This may reflect a situation where priming does occur for some subjects, which is in and of itself a finding worth pursuing further: IS may not be equally effective for all subjects, owing to individual differences in the combination of information from the two eyes (which is not involved in masking).

The absence of an unconscious repetition priming effect under IS is a bit surprising, as it was previously reported by at least two studies (Barbot and Kouider, 2011; Stein et al., 2013). In addition, it was shown that the similarity between two stimuli differing only by size (generally 20%, as in our repetition priming procedure) can be captured as early as the primary visual cortex (Faivre and Koch, 2014). We expected this similarity to potentially drive repetition priming under IS, which fell short of significance at the lowest mask contrast (*p* = 0.13). The absence of priming here may be attributed to the fact that our stimuli were very carefully equated in terms of low-level visual properties (a Gaussian mask removed peripheral facial features such as hair and ears as well as the background; faces were carefully aligned and matched for shape; image histograms were equated), which was not the case in previous studies. In addition, the absence of a significant effect may stem from the relatively smaller number of subjects, and fewer trials per subject that we collected in Experiment 1, hence making Experiment 1 (more) underpowered. This is because we were expecting to find strong effects in the repetition priming experiment and focused our resources on the identity priming (different viewpoint) experiment.

In our efforts to equate masking and IS we had to strip the latter down to a lesser version of what it really is. The decision to use one or the other technique is usually dictated by the duration of the stimulus that the researcher wishes to mask. If the researcher plans to mask a stimulus longer than about 50ms, then the combination of forward and backward masking becomes less effective and Continuous Flash Suppression (Tsuchiya and Koch, 2005) is usually preferred. For a fair comparison with masking, we are therefore limited to presentation time of the stimulus to 50 ms in IS (which we see as a lesser form of CFS). Under these brief presentation times, we found that face processing was qualitatively more disrupted than with masking, making a point on how the two techniques differ in their mechanisms and behavioral consequences. However, our data does not pertain to whether longer invisible stimulus durations as typically used with CFS would allow for higher level processing of faces, as previous reports have claimed. In the future, one could extend the comparison we performed to conditions of longer stimulus duration, for example using a modified version of masking allowing for longer (but still discontinuous) suppression periods like the standing wave of invisibility (Macknik and Livingstone, 1998).

We want to emphasize the potential importance of small, apparently harmless variations in experimental design. Here for instance, primes were clearly visible in as many as one third of the trials at the lowest mask contrast. Does intermixing visible trials with invisible trials influence the unconscious processing of invisible primes by changing subject's attention and expectations? Does the proportion of these visible trials matter? Further experiments would be needed to assess this empirically. While we report effects that paint masking as allowing more and higher level processing of masked stimuli than IS, we do not know whether these effects would hold if primes were invisible in all trials. Perhaps this is one source of the differences between our study and previous results on face identity priming (Henson et al., 2008; Kouider et al., 2009). Such idiosyncrasies that are difficult to faithfully adhere to when replicating an experiment from a published paper are the main reason why comparing different suppression techniques should be done in the context of a single experiment, in the hands of the same researcher, equating all parameters that can be equated. We encourage other researchers to conduct such controlled studies and are hopeful that a better understanding of unconscious processing, and guidelines on which technique is appropriate to disrupt which level of processing, will emerge from such an effort.

### Conflict of interest statement

The authors declare that the research was conducted in the absence of any commercial or financial relationships that could be construed as a potential conflict of interest.
