# Supplementary material for: A direct comparison of unconscious face processing under masking and interocular suppression
Source: Front Psychol. 2014 Jul 7;5:659. doi: 10.3389/fpsyg.2014.00659 (PMC4083827; doi:10.3389/fpsyg.2014.00659)
Supplement: Supplementary file 1 [file DataSheet1.DOCX]

***Supplementary Information***

**A direct comparison of unconscious face processing under masking and interocular suppression**

*Gregory Izatt^1†^, Julien Dubois^1†^*, Nathan Faivre^1,2†^, and Christof Koch ^1,3^*

*^†^these authors contributed equally*

^1^ Computation and Neural Systems, California Institute of Technology, Pasadena, CA, USA

^2^ Laboratory of Cognitive Neuroscience, Brain Mind Institute, School of Life Sciences, École Polytechnique Fédérale de Lausanne, 1015 Lausanne, Switzerland.

^3^ Allen Institute for Brain Science, Seattle, WA, USA

***Correspondence**: Julien Dubois, Division of Biology 114-96, California Institute of Technology, 1200 E California Blvd, Pasadena, CA 91125, [jcrdubois@gmail.com](mailto:jcrdubois@gmail.com)

Supplementary Table 1: Names of American “famous” females (FF) and famous males (FM), and Israeli “unfamiliar” females (UF) and males (UM), used in experiment.

| **FF** | *Christina Aguilera* | **UF** | *Tamar Ish-Shalom* |
| --- | --- | --- | --- |
| **FF** | *Drew Barrymore* | **UF** | *Ninet Tayeb* |
| **FF** | *Hillary Clinton* | **UF** | *Linoi Bar gefen* |
| **FF** | *Moley Cyrus* | **UF** | *Geula Even* |
| **FF** | *Sarah Palin* | **UF** | *Noa Tishbi* |
| **FF** | *Angelina Jolie* | **UF** | *Avigail Arieli* |
| **FF** | *Anna Hathaway* | **UF** | *Limor Buhadana* |
| **FF** | *Beyonce Knowles-Carter* | **UF** | *Esti Ginzburg* |
| **FF** | *Cameron Diaz* | **UF** | *Keren Peles* |
| **FF** | *Courteney Cox* | **UF** | *Tali Sharon* |
| **FF** | *Demi Moore* |  |  |
| **FF** | *Ellen DeGeneres* |  |  |
| **FF** | *Vanna White* |  |  |
| **FF** | *Britney Spears* |  |  |
| **FF** | *Hayley Mills* |  |  |
| **FF** | *Halle Berry* |  |  |
| **FF** | *Helen Hunt* |  |  |
| **FF** | *Jennifer Lopez* |  |  |
| **FF** | *Jennifer Aniston* |  |  |
| **FF** | *Julia Roberts* |  |  |
| **FF** | *Julia Louis-Dreyfus* |  |  |
| **FF** | *Katie Holmes* |  |  |
| **FF** | *Kim Kardashian* |  |  |
| **FF** | *Madonna Ciccone* |  |  |
| **FF** | *Michelle Obama* |  |  |
| **FF** | *Natalie Portman* |  |  |
| **FF** | *Nicole Kidman* |  |  |
| **FF** | *Oprah Winfrey* | **UM** | *Sasi Keshet* |
| **FF** | *Rihanna Fenty* | **UM** | *Oz Zehavi* |
| **FF** | *Shakira Ripoll* | **UM** | *Ivri Lider* |
| **FF** | *Sharon Stone* | **UM** | *Guy Zilberman* |
| **FM** | *Arnold Schwarzenegger* | **UM** | *Asi Cohen* |
| **FM** | *Bill Clinton* | **UM** | *Lior Ashkenazi* |
| **FM** | *Ryan Seacrest* | **UM** | *Oshri Cohen* |
| **FM** | *George Clooney* | **UM** | *Yehuda Levi* |
| **FM** | *Jay Leno* | **UM** | *Guy Arieli* |
| **FM** | *Jerry Seinfeld* | **UM** | *Rami Klienstein* |
| **FM** | *Keanu Reeves* |  |  |
| **FM** | *Leonardo Dicaprio* |  |  |
| **FM** | *Tom Cruise* |  |  |
| **FM** | *Adam Sandler* |  |  |
| **FM** | *George W. Bush* |  |  |
| **FM** | *Barack Obama* |  |  |
| **FM** | *Ben Stiller* |  |  |
| **FM** | *Brad Pitt* |  |  |
| **FM** | *Bruce Willis* |  |  |
| **FM** | *David Letterman* |  |  |
| **FM** | *Harrison Ford* |  |  |
| **FM** | *Hugh Grant* |  |  |
| **FM** | *Jim Carrey* |  |  |
| **FM** | *Johnny Depp* |  |  |
| **FM** | *Justin Timberlake* |  |  |
| **FM** | *Justin Bieber* |  |  |
| **FM** | *Mel Gibson* |  |  |
| **FM** | *Nicolas Cage* |  |  |
| **FM** | *Pierce Brosnan* |  |  |
| **FM** | *Robin Williams* |  |  |
| **FM** | *Russell Crowe* |  |  |
| **FM** | *Ryan Gosling* |  |  |
| **FM** | *Tom Hanks* |  |  |
| **FM** | *Will Smith* |  |  |

Supplementary Table 2: The number of trials taken from each subject in Experiment 1. Subjects with 0 total trials were excluded for having a main task accuracy below 70%.

|  | | **All Subj. Vis.** | | **Subj. Vis. 1-2** | |
| --- | --- | --- | --- | --- | --- |
| **Subj #** | **Total Trials** | **Analyzed Trials** | **% Trials Kept** | **Analyzed Trials** | **% Trials Kept** |
| 1 | 720 | 654 | 90.83% | 432 | 60.00% |
| 2 | 720 | 618 | 85.83% | 0 | 0.00% |
| 3 | 720 | 556 | 77.22% | 330 | 45.83% |
| 4 | 576 | 494 | 85.76% | 425 | 73.78% |
| 5 | 432 | 349 | 80.79% | 345 | 79.86% |
| 6 | 0 | 0 | 0.00% | 0 | 0.00% |
| 7 | 720 | 646 | 89.72% | 411 | 57.08% |
| 8 | 720 | 619 | 85.97% | 355 | 49.31% |
| 9 | 720 | 649 | 90.14% | 613 | 85.14% |
| 10 | 432 | 411 | 95.14% | 0 | 0.00% |
| 11 | 720 | 625 | 86.81% | 298 | 41.39% |
| 12 | 720 | 560 | 77.78% | 560 | 77.78% |
| 13 | 0 | 0 | 0.00% | 0 | 0.00% |
| 14 | 720 | 637 | 88.47% | 414 | 57.50% |
| 15 | 0 | 0 | 0.00% | 0 | 0.00% |
| 16 | 720 | 651 | 90.42% | 594 | 82.50% |
| 17 | 576 | 536 | 93.06% | 494 | 85.76% |
| 18 | 576 | 520 | 90.28% | 318 | 55.21% |
|  | | *MEAN:* | 72.68% | *MEAN:* | 47.29% |

Supplementary Table 3: The number of trials taken from each subject in Experiment 2. Subjects with 0 total trials were excluded for having a main task accuracy below 70%.

|  | | **All Subj. Vis.** | | **Subj. Vis. 1-2** | |
| --- | --- | --- | --- | --- | --- |
| **Subj #** | **Total Trials** | **Analyzed Trials** | **Fract. Trials Kept** | **Analyzed Trials** | **Fract. Trials Kept** |
| 1 | 720 | 523 | 72.64% | 460 | 63.89% |
| 2 | 720 | 565 | 78.47% | 560 | 77.78% |
| 3 | 720 | 644 | 89.44% | 516 | 71.67% |
| 4 | 720 | 346 | 48.06% | 320 | 44.44% |
| 5 | 720 | 539 | 74.86% | 477 | 66.25% |
| 6 | 720 | 632 | 87.78% | 563 | 78.19% |
| 7 | 720 | 663 | 92.08% | 532 | 73.89% |
| 8 | 0 | 0 | 0.00% | 0 | 0.00% |
| 9 | 720 | 633 | 87.92% | 340 | 47.22% |
| 10 | 0 | 0 | 0.00% | 0 | 0.00% |
| 11 | 720 | 662 | 91.94% | 581 | 80.69% |
| 12 | 720 | 627 | 87.08% | 582 | 80.83% |
| 13 | 720 | 573 | 79.58% | 500 | 69.44% |
| 14 | 720 | 662 | 91.94% | 391 | 54.31% |
| 15 | 720 | 629 | 87.36% | 323 | 44.86% |
| 16 | 720 | 620 | 86.11% | 609 | 84.58% |
| 17 | 720 | 533 | 74.03% | 529 | 73.47% |
| 18 | 720 | 608 | 84.44% | 506 | 70.28% |
| 19 | 720 | 614 | 85.28% | 613 | 85.14% |
| 20 | 720 | 627 | 87.08% | 616 | 85.56% |
| 21 | 720 | 643 | 89.31% | 0 | 0.00% |
| 22 | 720 | 645 | 89.58% | 644 | 89.44% |
| 23 | 720 | 623 | 86.53% | 616 | 85.56% |
| 24 | 720 | 641 | 89.03% | 577 | 80.14% |
| 25 | 720 | 585 | 81.25% | 557 | 77.36% |
| 26 | 720 | 570 | 79.17% | 564 | 78.33% |
|  | | *MEAN:* | 76.96% | *MEAN:* | 63.97% |


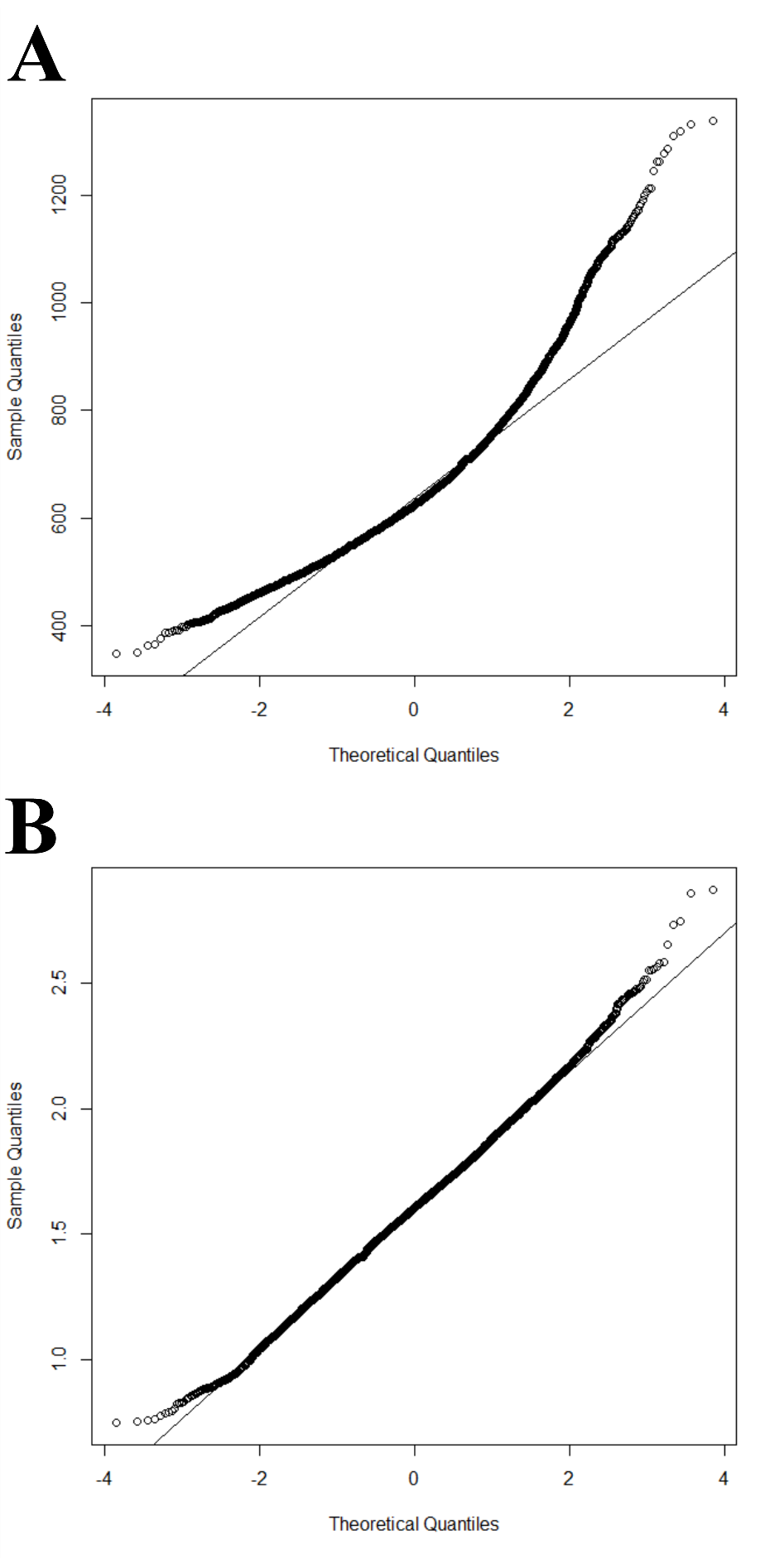

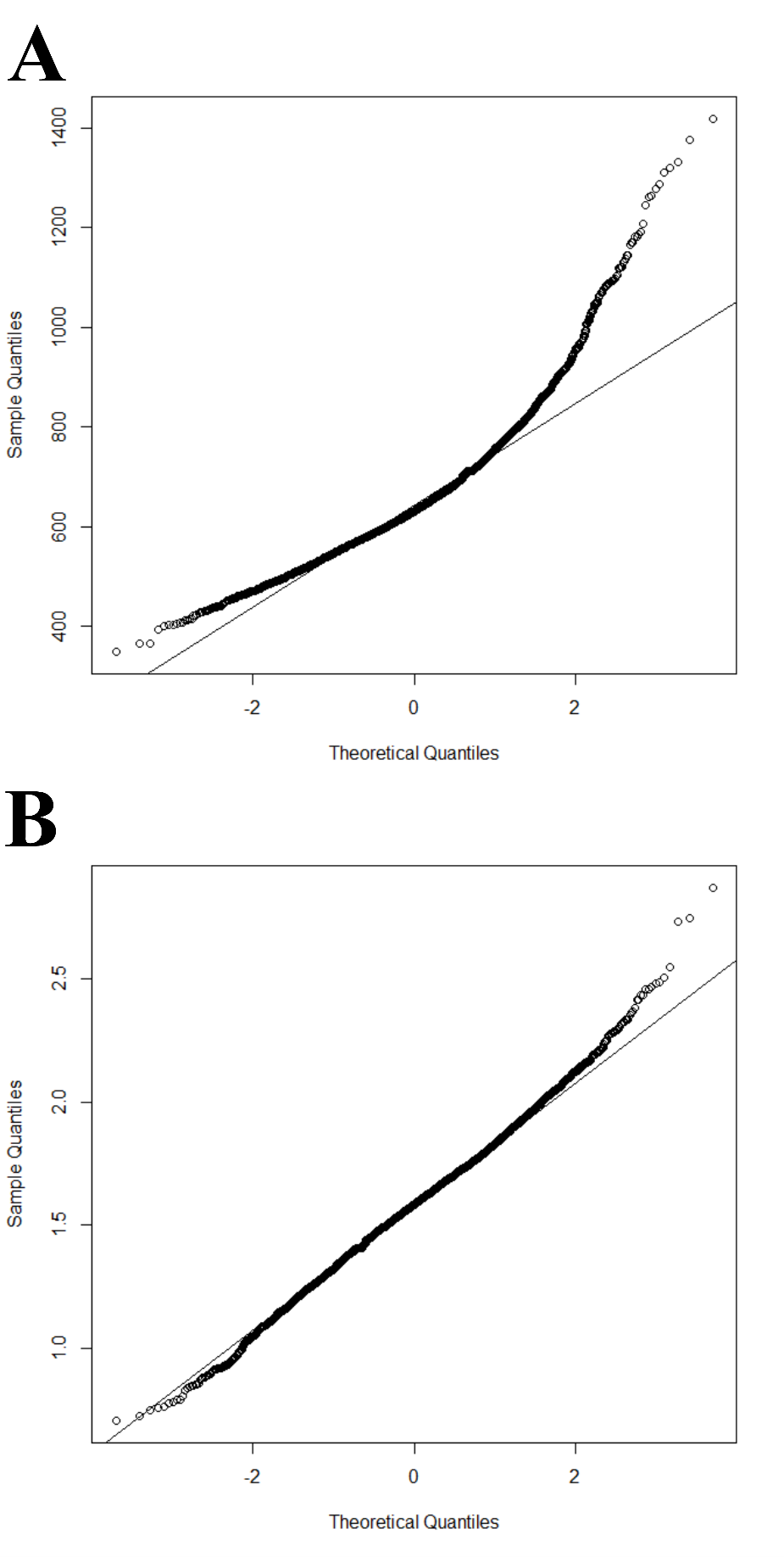

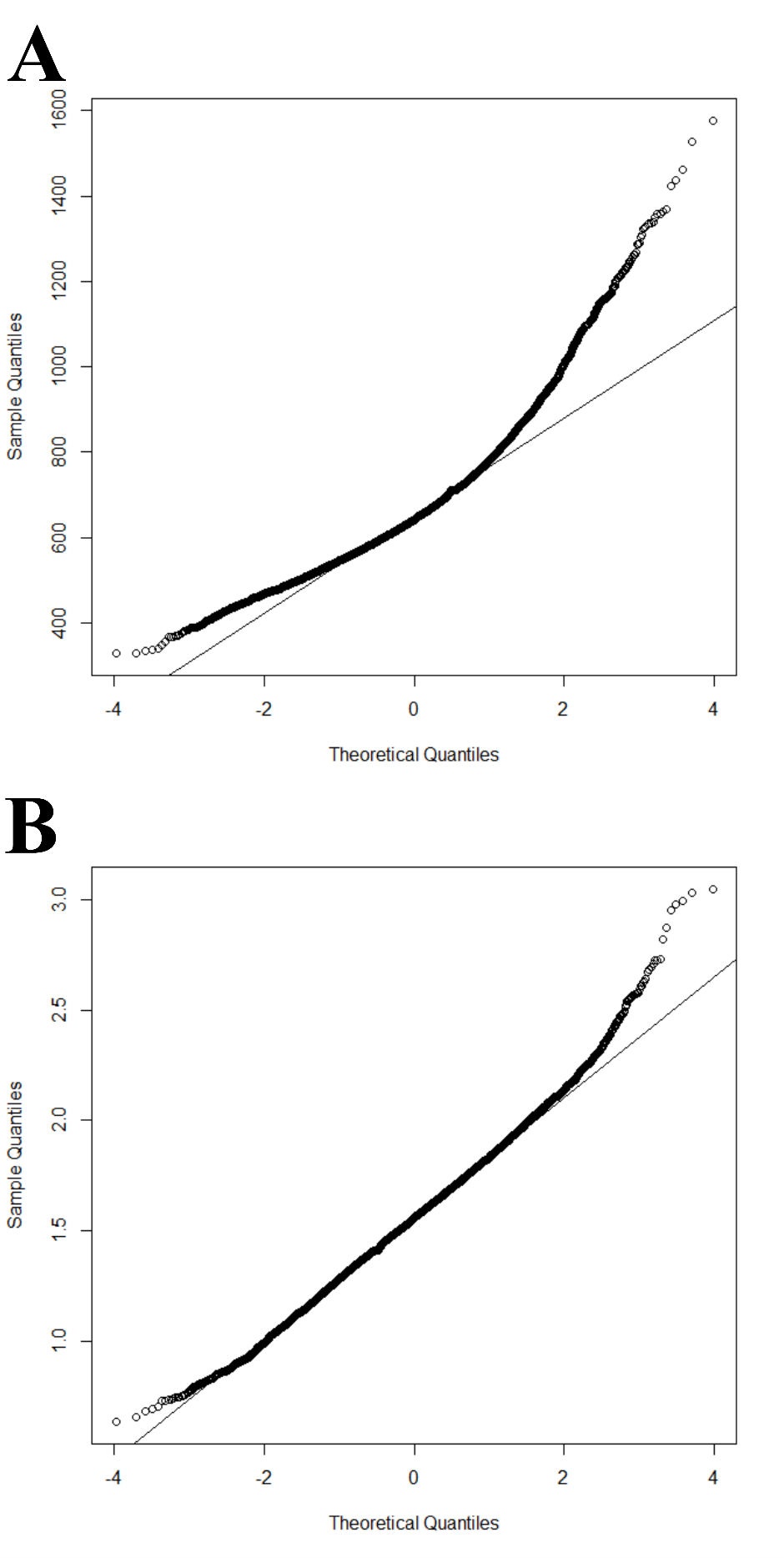


Supplementary Figure 1: **(A)** Q-Q plot of untransformed trials from Experiment 1 (without selection by subjective visibility).

**(B)** Q-Q plot of the same trials under an inverse transform.

Supplementary Figure 2: **(A)** Q-Q plot of untransformed trials from Experiment 1 (selecting only those trials with subjective visibility rating 1 or 2, and excluding trials with the lowest mask contrast).

**(B)** Q-Q plot of the same trials under an inverse transform.

Supplementary Figure 3: **(A)** Q-Q plot of untransformed trials from Experiment 2 (without selection by subjective visibility rating).

**(B)** Q-Q plot of the same trials under an inverse transform.


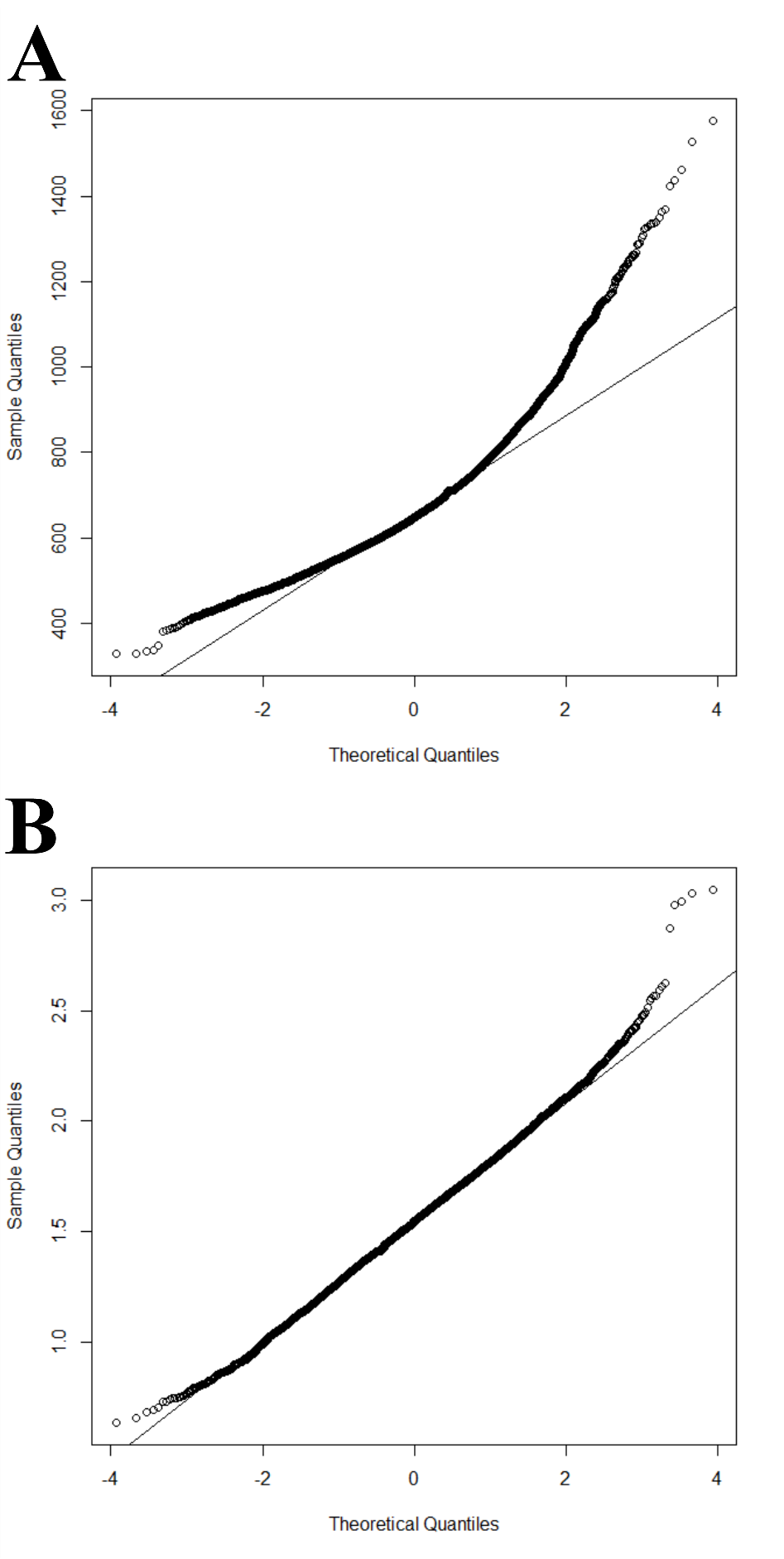


Supplementary Figure 4: **(A)** Q-Q plot of untransformed trials from Experiment 2 (selecting only those trials with subjective visibility rating 1 or 2).

**(B)** Q-Q plot of the same trials under an inverse transform.


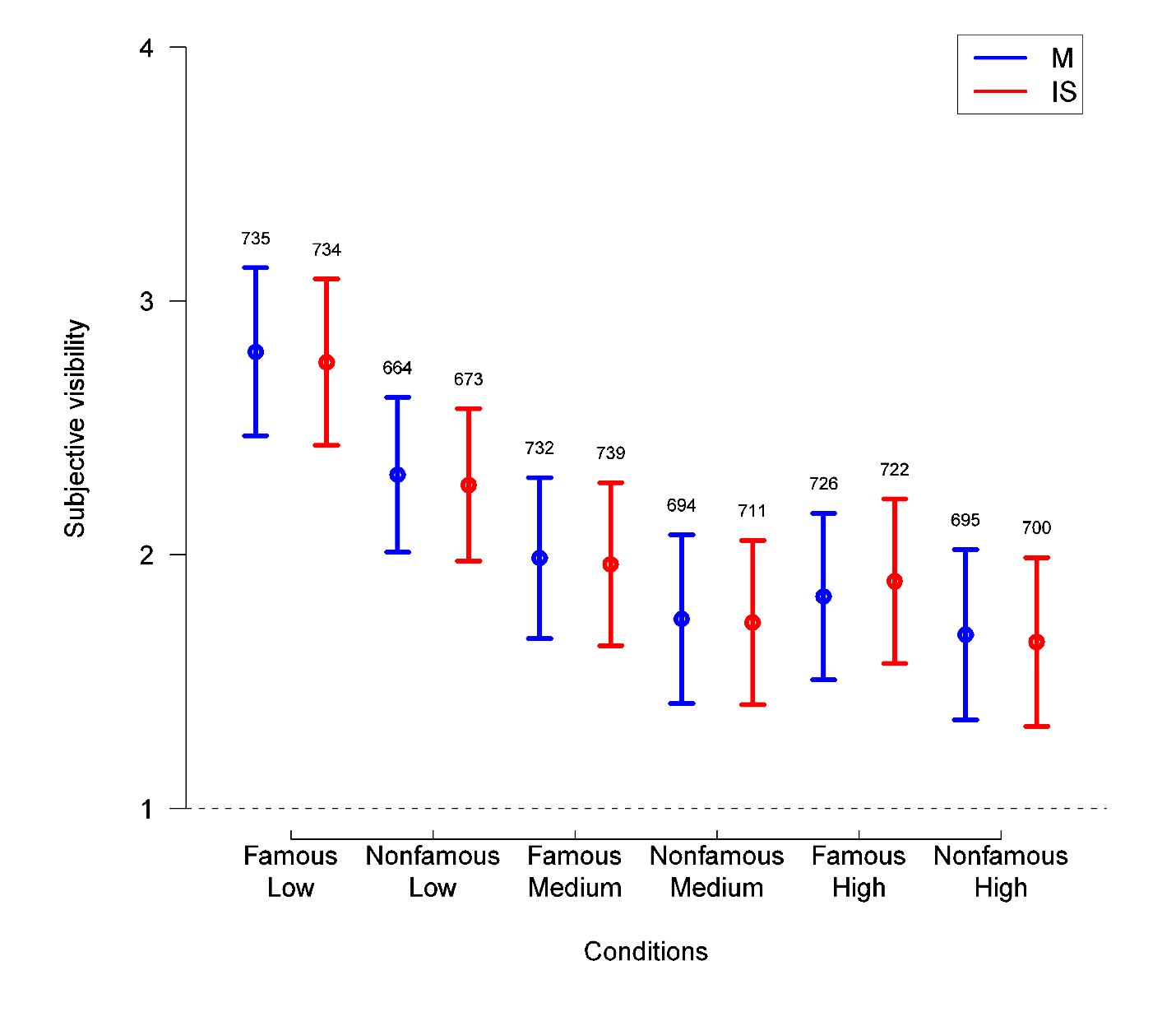


Supplementary Figure 5: Subjective visibility ratings observed in Experiment 1, for famous and unfamiliar faces at different mask contrast levels (low, medium and high) and masking methods (M and IS), with no selection of trials based on subjective visibility rating (i.e. all trials are shown). Also listed for each category are the number of trials analyzed in that condition.

**
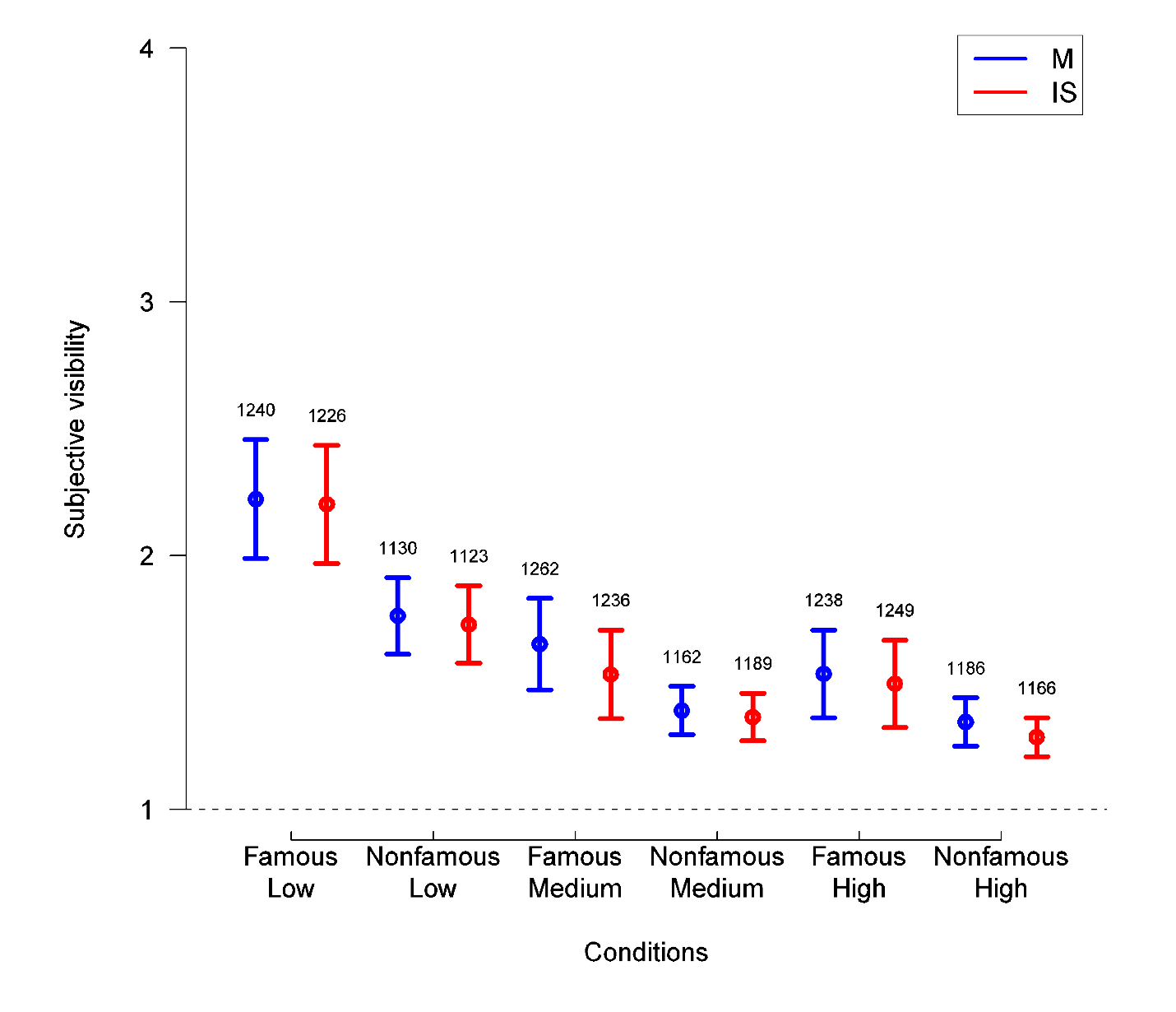
**Supplementary Figure 6: Subjective visibility ratings observed in Experiment 2, for famous and unfamiliar faces at different mask contrast levels (low, medium and high) and masking methods (M and IS), with no selection of trials based on subjective visibility rating (i.e. all trials are shown). Also listed for each category are the number of trials analyzed in that condition.
